# An Improved A-Star Path Planning Algorithm Based on Mobile Robots in Medical Testing Laboratories

**DOI:** 10.3390/s24061784

**Published:** 2024-03-10

**Authors:** Chengpeng Yin, Chunyu Tan, Chongqin Wang, Feng Shen

**Affiliations:** 1 School of Mechanical and Electrical Engineering, Harbin Engineering University, Harbin 150001, China; 2 School of Intelligent Science and Engineering, Harbin Engineering University, Harbin 150001, China; 3 School of Electrical and Electronic Engineering, Harbin University of Science and Technology, Harbin 264300, China; 4 School of Instrumentation Science and Engineering, Harbin Institute of Technology, Harbin 150001, China

**Keywords:** A-star algorithm, path planning, bi-directional search, path smoothing

## Abstract

In the blood sample management pipeline environment, we have innovatively improved the traditional A-star algorithm to enhance the efficiency of mobile robots. This study employs a grid environmental modeling approach to accurately simulate medical testing laboratories. On the grid map, we utilize an 8-neighbor search method for path planning to accommodate the complex structure within the laboratory. By introducing an improved evaluation function and a bidirectional search strategy, we have successfully reduced the number of search nodes and significantly improved path search efficiency. Additionally, we eliminate redundant nodes in the path, smooth the path using cubic uniform B-spline curves, remove unnecessary inflection points, and further optimize the motion trajectory of the robot. The experimental results of the path planning simulation under different scenarios and specifications show that the improved A-star algorithm has higher search efficiency and traverses fewer nodes compared to the traditional A-star algorithm and the bidirectional A-star algorithm. Overall, the simulation experiments verify the feasibility of the improved A-star algorithm, which can better meet the needs of actual mobile robots in real medical testing laboratories.

## 1. Introduction

In recent years, under the guidance of the “Healthy China” strategy, the state has issued a number of policies requiring medical institutions to improve the level of intelligence and further improve the patient medical experience. Blood sample testing and management play a crucial role in healthcare organizations. Therefore, optimization of blood sample testing and management is an essential component of achieving intelligent healthcare. Blood sample testing and management heavily relies on manual operations, which are repetitive and cumbersome tasks. The introduction of autonomous mobile robots can effectively eliminate direct contact between medical staff and blood samples, freeing them from repetitive tasks and saving more time to devote to patient care.

Path planning is a crucial step for autonomous mobile robots to achieve intelligent decision-making. Path planning is the process of using algorithms and strategies to determine the optimal path from a start point to a goal point in a given environment [1,2]. Path planning is mainly divided into local path planning and global path planning. Common global path planning algorithms include the Dijkstra algorithm [3], the A-star algorithm [4], the RRT algorithm [5] and the artificial potential field method [6], etc. The A-star algorithm has become one of the most widely used global path planning algorithms due to its ease of implementation, high search efficiency, and wide applicability. In the environment of the blood sample management assembly line, the requirements for autonomous mobile robots prioritize speed, accuracy, and stability. As a result, we have selected the A-star algorithm as the path planning algorithm for autonomous mobile robots. However, the traditional A-star algorithm suffers from a high number of search nodes, a high number of redundant nodes in the generated path, and poor path smoothing. These issues not only impact the stability of autonomous mobile robots but also decrease the efficiency of path planning.

Therefore, we still need to improve the A-star algorithm to avoid the above problems. In recent years, both domestic and international researchers have made significant progress in modifying the A-star algorithm. To address the issue of a high number of search nodes and redundant nodes in the generating path, Haoxin Liu et al. [7] improved the evaluation function of the A-star algorithm and successfully reduced the number of search nodes. Yifan Wang et al. [8] designed a redundancy evaluation function and used an adaptive variable step size strategy to reduce the number of expansion nodes, successfully eliminating redundant nodes in the path. Jing Zhang et al. [9] improved the evaluation function of the A-star algorithm and combined it with the artificial potential field method to effectively reduce the number of redundant nodes in the path. However, the introduction of the artificial potential field method increases the computational complexity and has a large storage space requirement. Tang et al. [10] optimized the irregular path by screening the function and finally smoothed the entire path using an interpolation algorithm. Erke et al. [11] improved obstacle avoidance performance by adopting the idea of critical points, in addition to obstacles, while proposing an A-star algorithm based on variable step size. To address the issue of poor path smoothness, Yibo Li et al. [12] utilized the tangent method to optimize the path at inflection points, effectively shortening the path length. However, the applicability of the tangent method is limited, and the parameters need to be adjusted based on different scenarios and robot characteristics.

Based on the advantages and shortcomings of the situation of autonomous mobile robots running the A-star algorithm in the blood sample specimen management pipeline environment, this paper proposes an improved A-star algorithm with the following main elements:

1. Based on the evaluation function of the traditional A-star algorithm, the heuristic function is improved by introducing the angle and scale factor of the line connecting the node to the start point and the goal point.
2. We adopt the bidirectional search strategy to compute the coordinates of the intermediate point from the coordinates of the start and the goal points, and at the same time traverse the nodes from the intermediate point to the start and the goal points.
3. We optimize the path to remove redundant nodes by judging the intersection of the links between nodes with obstacles.
4. After removing redundant nodes, the cubic uniform B-spline curve is introduced to smooth the path.

Finally, simulation experiments verify the feasibility of the proposed method and its adaptability in different scenarios.

## 2. Conventional A-Star Algorithm

### 2.1. Establishment of Environment Model

Before path planning, the robot needs to determine the environment information and build a map model. The map model is crucial for the path planning algorithm, which directly affects the navigation and decision-making capabilities of autonomous mobile robots in complex environments. Environmental modeling commonly includes grid maps [13], Voronoi maps [14], and topological maps, etc. This experiment uses the grid map for environment modeling. Grid maps, a widely used representation for robot path planning, divide the environment into regular grid cells, each representing a discrete region on the map. The experiment marks passable areas as free grids, shown in white, and inaccessible areas as obstacle grids, shown in black. This approach reduces the level of abstraction of the complex environment and expresses the structure of the environment in an intuitive and simple way, which helps to reduce the complexity of the path planning problem and improve the efficiency of the A-star algorithm in the search space.

The accuracy of the environment map is mainly determined by the size of the grid cells. The smaller the grid cell size, the more accurately details of the environment can be captured, including the shape and location of obstacles [15]. As the size of the grid cell decreases, the amount of data in the environment map increases proportionally, resulting in more memory resources to be occupied. Therefore, in order for the robot to avoid obstacles and find the optimal path more efficiently, it is necessary to select the appropriate size of grid cells.

**Figure 1:**
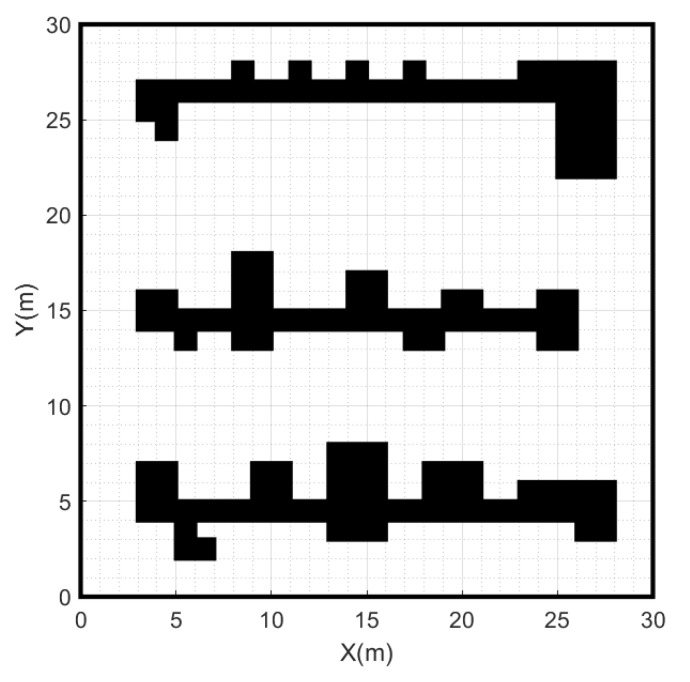
Environmental map.

Based on the MATLAB platform, we used the grid map method to generate a 30 × 30 raster map, where each grid cell has a side length of 1, as shown in Figure 1.

### 2.2. Search Neighborhood Selection

**Figure 2:**
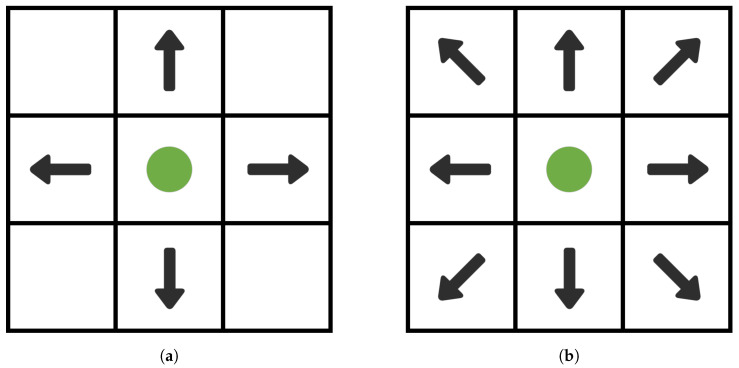
Diagram of neighborhood search method. (**a**) 4-neighborhood search, (**b**) 8-neighborhood search.

In the environmental map, the choice of search neighborhood is crucial when the A-star algorithm traverses the nodes from the start point [16,17]. Search neighborhood selection is the process of selecting a goal node within a specified area around the current node. Common search neighborhood selection methods include 4-neighborhood search and 8-neighborhood search, as shown in Figure 2.

4-neighborhood search is centered on the current node and searches along the four directions of up, down, left, and right. This strategy has a simple search process and relatively fast computation, but it may not take into account the complexity of the current environment. The 8-neighborhood search is an extension of the 4-neighborhood search that includes four diagonal directions: top-left, bottom-left, top-right, and bottom-right. This strategy enables a more comprehensive exploration of the surrounding environment, increasing the flexibility and accuracy of the search. Therefore, this paper uses the 8-neighborhood search method.

### 2.3. Principle of Traditional A-Star Algorithm

The A-star algorithm is a heuristic search algorithm that expands nodes in the domain from the start point. It selects the node with the smallest cost to expand by comparing the surrogate values of neighboring nodes until it reaches the goal point. Thus, it avoids obstacles and finds the optimal path from the start point to the goal point. Its evaluation function is

(1) f(n)=g(n)+h(n)

f(n) — The combined priority of node n. When selecting the next node to traverse, we always select the node with the smallest value.

g(n) — The actual cost of node n to the start point.

h(n) — The heuristic cost of node n to the goal point.

In the A-star algorithm, the combined priority of the nodes depends on the actual path cost g(n) and the heuristic estimated cost h(n). When g(n) is much larger than h(n), the priority of the node is mainly determined by the actual path cost g(n). In this case, the A-star algorithm, which prioritizes breadth-first search, expands the domain nodes layer by layer from the start point. Thus, the search space is increased, ignoring the role of the heuristic function in guiding the search direction. On the contrary, when the heuristic estimation cost h(n) is much larger than the actual path cost g(n), the priority of the nodes is mainly determined by the actual path cost h(n). In this case, the A-star algorithm prioritizes depth-first search. It explores the path along the depth direction of the graph from the start point until it reaches the deepest point, and then backtracks to the previous layer to continue exploring in depth, rather than prioritizing the direction of the potentially optimal solution.

**Figure 3:**
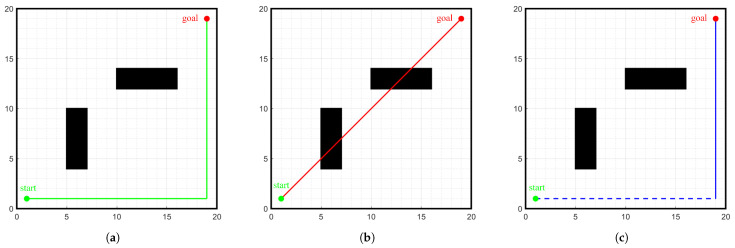
Diagram of the heuristic function. (**a**) Manhattan distance, (**b**) Euler distance, (**c**) Chebyshev distance.

The commonly used heuristic functions for the A-star algorithm include Manhattan distance, Euler distance, and Chebyshev distance, as shown in Figure 3.

The Manhattan distance formula is

(2) $$h\left(n\right)=|x_{n}-x_{\mathrm{goal}}\left|+\right|y_{n}-y_{\mathrm{goal}}|$$

The Euler distance formula is

(3) $$h\left(n\right)=\sqrt{\left({\left(x_{n}-x_{\mathrm{goal}}\right)}^{2}+{\left(y_{n}-y_{\mathrm{goal}}\right)}^{2}\right)}$$

The Chebyshev distance formula is

(4) $$h\left(n\right)=max(|x_{n}-x_{\mathrm{goal}}|,|y_{n}-y_{\mathrm{goal}}|)$$

where,$\left(x_{n},y_{n}\right)$ is the current node, and $\left(x_{\mathrm{goal}},y_{\mathrm{goal}}\right)$ is the goal point.

On the environment map shown in Figure 1, path planning is conducted from the start point (1, 1) to the goal point (29, 29) using different heuristic functions h(n) and the 8-neighborhood search method. By analyzing the experimental results, it can be found that the heuristic function h(n) calculated using Manhattan distance reduces the number of nodes and turns, as well as the time required to plan the path. The specific data are shown in Table 1. Thus, this paper selects the Manhattan distance as the cost function for the heuristic function h(n).

## 3. Improved A-Star Algorithm

### 3.1. Improving the Evaluation Function

The traditional A-star algorithm only considers the distance problem and does not take into account many practical factors on the environment map. Based on the evaluation function of the traditional A-star algorithm, we improve the evaluation function by introducing the angle between the node and the line that connects the start point and the goal point [18].

**Figure 4:**
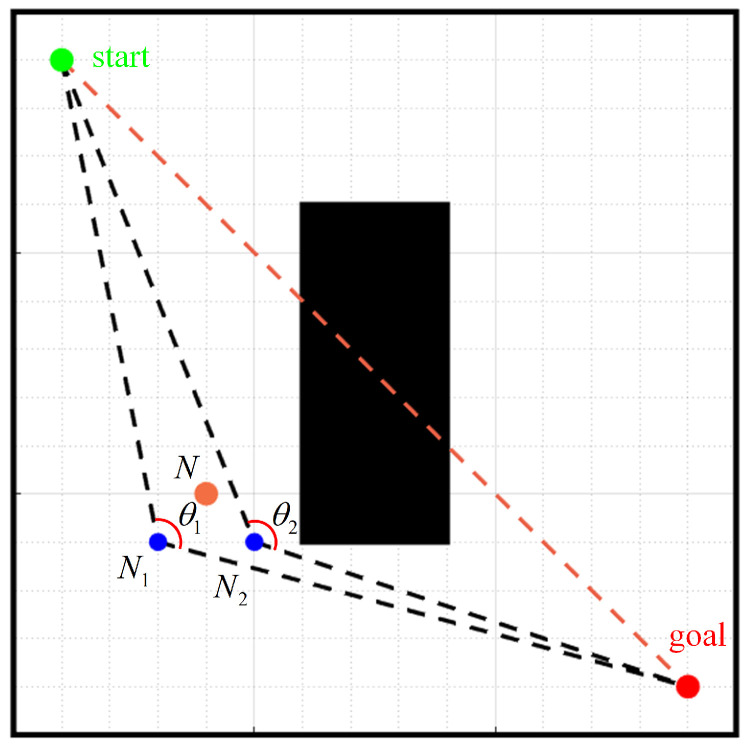
Diagram of the node angle.

As shown in Figure 4, the green point is the start point $\left(x_{\mathrm{start}},y_{\mathrm{start}}\right)$, the red point is the goal point $\left(x_{\mathrm{goal}},y_{\mathrm{goal}}\right)$, and the blue points are the two neighbor nodes $N_{1}$$\left(x_{1},y_{1}\right)$ and $N_{2}$$\left(x_{2},y_{2}\right)$ of node *N*.

The current node $N_{1}$ is connected to the start point and the goal point, respectively. The two vectors are

(5) $$v1=[x_{\mathrm{start}}-x_{1},y_{\mathrm{start}}-y_{1}]$$

(6) $$v2=[x_{\mathrm{goal}}-x_{1},y_{\mathrm{goal}}-y_{1}]$$

The angle θ between two vectors is

(7) $$\theta =\arccos\left(\frac{v_{1}\cdot v_{2}}{∥v_{1}∥∥v_{2}∥}\right)$$

The calculation formula of c(n) is

(8) $$c\left(n\right)=3.14-\arccos\left(\frac{v_{1}\cdot v_{2}}{∥v_{1}∥∥v_{2}∥}\right)$$

Thus, the evaluation function of the current node *n* is

(9) f(n)=g(n)+h(n)+μ×c(n)

where, f(n), g(n), and h(n) are defined as in Formula (1). c(n) denotes the cost of the angle between the current node and the line connecting the start and goal points. μ represents the scale factor.

The new evaluation function adds c(n), whose purpose is to make the A-star algorithm search for nodes as close as possible to the line connecting the start point and the goal point when traversing the nodes. The larger the value of θ, the closer it is to the straight line, and the smaller the value of c(n), the closer it is to the connecting line when traversing the nodes, thereby reducing the number of search nodes and improving the search efficiency.

### 3.2. Introducing the Bidirectional Search Strategy

The traditional A-star algorithm adopts a unidirectional search strategy in large search spaces. However, this method can result in low search efficiency and a high number of search nodes. Thus, we introduce the midpoint $\left(x_{\mathrm{mid}},y_{\mathrm{mid}}\right)$. Its coordinate calculation formula is

(10) $$x_{\mathrm{mid}}=\frac{x_{\mathrm{start}}+x_{\mathrm{goal}}}{2}$$

(11) $$y_{\mathrm{mid}}=\frac{y_{\mathrm{start}}+y_{\mathrm{goal}}}{2}$$

A bidirectional search strategy is used, where the midpoint is used as the start point of the search, and the search is initiated simultaneously from the intermediate point to the start and goal points. This method divides the search space into two independent regions, which reduces the search size in each direction and enhances the overall search efficiency.

**Figure 5:**
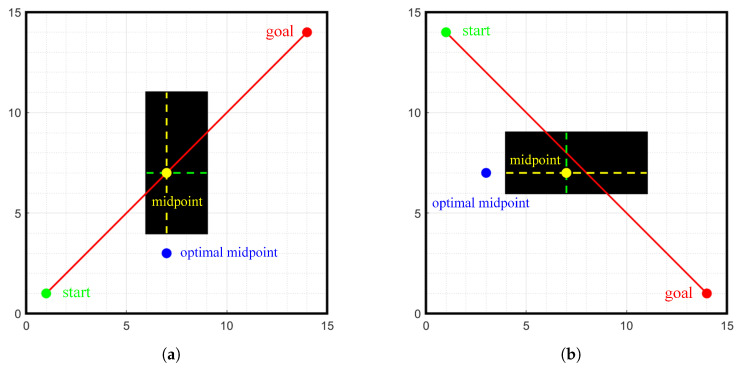
Diagram of the new midpoint selection strategy. (**a**) vertical direction, (**b**) horizontal direction.

When the calculated midpoint is inside an obstacle, it prevents an effective search from the midpoint to the start and goal points. Thus, we need to select the new midpoint, as shown in Figure 5. The specific steps are as follows:

Step 1: Determine the position of the midpoint.
Step 2: Draw a horizontal line and a vertical line at the midpoint position.
Step 3: Calculate the length of horizontal and vertical lines when crossing obstacles.
Step 4: Select the direction with the longer crossing length and, in that direction, find the earliest point to cross the obstacle.
Step 5: Select the earliest point that crosses the obstacle as the optimized midpoint.

The strategy outlined above ensures the effectiveness of the midpoint selection and enhances the search algorithm’s ability to adapt to various environments, thus improving the adaptability and robustness of this search algorithm.

### 3.3. Removing Redundant Nodes

In the grid map, we use the Improved A-star algorithm to compute and retain each grid node, which is then connected into a path. However, there are many redundant nodes in the path, which can cause problems such as excessive memory usage and frequent robot motion adjustments. To address this issue, we propose a path optimization method to remove redundant nodes.

**Figure 6:**
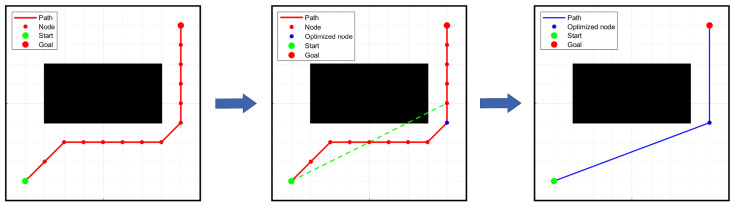
Diagram of removing redundant nodes.

As shown in Figure 6, the specific steps for removing redundant nodes are as follows:

Step 1: Use the start point as the start node.
Step 2: Along the path, connect the start point to each node in the path one by one.
Step 3: If the connecting line segment intersects an obstacle, the previous node of the intersecting node is retained and considered as the next start node, and all nodes between that node and the previous start node are removed. Then, return to step two.
Step 4: If the connecting line segment does not intersect any obstacles until it reaches the goal point, the goal point is retained and all nodes between the start node and the goal point are removed.
Step 5: Connect the retained nodes in the path sequentially to form a new path.

### 3.4. Smoothing the Planned Path

On the grid map, the path generated by the A-star algorithm suffers from issues such as the tendency to form sharp points at the inflection point and unsmooth path. This may result in inefficient turning and accumulation of movement errors during the actual movement of the autonomous mobile robot. To address this issue, we introduce a path smoothing strategy.

**Figure 7:**
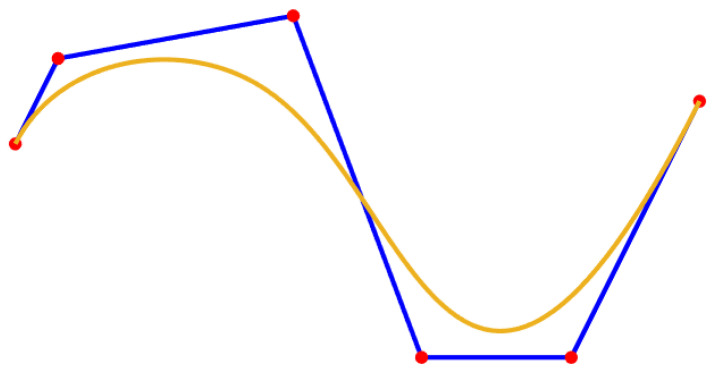
Diagram of the B-spline curve.

Commonly used smoothing path methods include B spline curves [19] and Bezier curves. The B-spline curve is a general form of the Bezier curve, as shown in Figure 7. The order and the number of control points of the B-spline curve can be varied independently, which has good local control and path smoothing effect [20]. Therefore, this paper selects the B-spline curve to smooth the path after removing redundant nodes.

The mathematical formulation of the B-spline curve is

(12) $$p\left(t\right)=\sum_{i=0}^{n}P_{i}N_{i,k}\left(t\right)$$

where, $P_{i}\left\{i=0,1,2,\ldots ,n\right\}$ is the characteristic point of the control curve, $N_{i,k}\left(t\right)$ is the basis function of the k-order B-spline curve, and k represents the order of the B-spline curve. n represents the number of control points, while t is the value used for plotting the curve.

The formula for the B-spline curve’s deBoor-Cox recursive definition is

(13) $$N_{i,k}\left(t\right)=\left\{\begin{array}{c} N_{i,1}\left(t\right)=\left\{\begin{array}{cc} 1 & t_{i}\leq t<t_{i+1}\\ 0 & Otherwise \end{array}\right.\\ N_{i,k}\left(t\right)=\frac{\left(t-t_{i}\right)}{\left(t_{i+k-1}-t_{i}\right)}N_{i,k-1}\left(t\right)+\frac{\left(t_{i+k}-t\right)}{\left(t_{i+k}-t_{i+1}\right)}N_{i+1,k-1}\left(t\right) \end{array}\right.$$

where, the convention 0/0=0 and $t_{i}=\left\{t_{i},t_{\left(i+1\right)},\ldots ,t_{\left(i+k\right)}\right\}$, and the interval $\left[t_{i},t_{\left(i+k\right)}\right]$ is called the support interval of $N_{i,k}\left(t\right)$.

When considering computational efficiency and cost, quadratic or cubic B-spline curves are commonly used. In addition, the uniform B-spline curve enables flexible control of the length and shape of the curve. Therefore, in this paper, the path obtained by the improved A-star algorithm in the grid map is smoothed by using cubic uniform B-spline curves.

The cubic uniform B-spline curve equation is

(14) $$p\left(t\right)=\sum_{i=0}^{n}P_{i}N_{i,3}\left(t\right)$$

Its basis function expression is

(15) $$\left\{\begin{array}{cc} N_{0,3}\left(t\right)=\frac{1}{6}\left(-t^{3}+3t^{2}-3t+1\right) & \\ N_{1,3}\left(t\right)=\frac{1}{6}\left(3t^{3}-6t^{2}+4\right) & \\ N_{2,3}\left(t\right)=\frac{1}{6}\left(-3t^{3}+3t^{2}+3t+1\right) & \\ N_{3,3}\left(t\right)=\frac{1}{6}t^{3} & \end{array}\right.$$

where, t∈[0,1]

### 3.5. Process and Pseudo-Code of the Improved A-Star Algorithm

**Figure 8:**
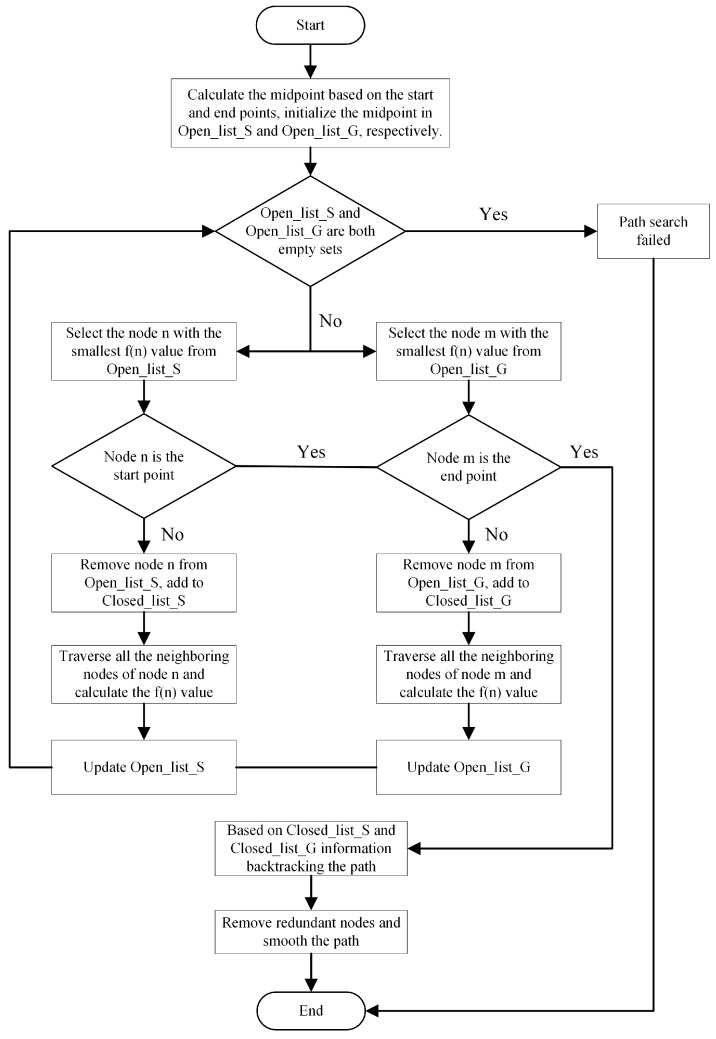
Diagram of the process of improving the A-star algorithm.

Based on the traditional A-star algorithm, we introduce a bidirectional search strategy, improve the heuristic function, remove the redundant nodes, and smooth the generated paths by using a cubic uniform B-spline curve, and the specific steps are shown in Figure 8.

## 4. Simulation Experiment and Analysis

To verify the feasibility and optimization effect of the improved algorithms proposed in this paper, we conduct simulation experiments on different algorithms and compare and analyse their results. The same experiment was repeated several times to ensure the accuracy of the results. The computer configuration used for the experiments in this paper is as follows: Windows 11 operating system, AMD Ryzen 7 5800H processor, 3.20 GHz main frequency, and 16 GB RAM.

### 4.1. Path Planning Simulation Experiment

**Figure 9:**
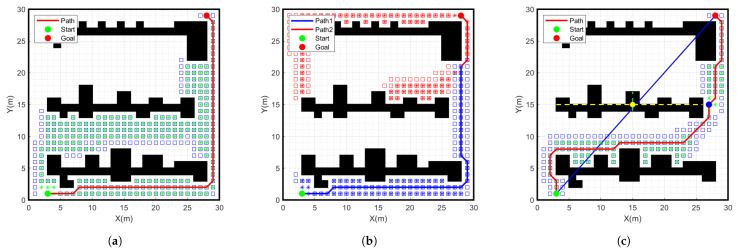
Simulation results of different algorithms for start point (3, 1) goal point (28, 29) on a 30 × 30 grid map. (**a**) Traditional A-star algorithm, (**b**) Bidirectional A-star algorithm, (**c**) Improved A-star algorithm.

**Figure 10:**
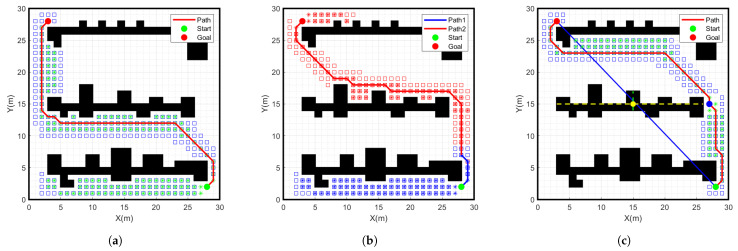
Simulation results of different algorithms for start point (28, 2) goal point (3, 28) on a 30 × 30 grid map. (**a**) Traditional A-star algorithm, (**b**) Bidirectional A-star algorithm, (**c**) Improved A-star algorithm.

The path planning simulation experiments were conducted in MATLAB 2020b by creating a 30 × 30 grid map and setting the start point as a green point and the goal point as a red point. In the experiments, we used three different path planning algorithms, which are the traditional A-star algorithm, the bidirectional A-star algorithm, and the improved A-star algorithm. As shown in Figure 9 and Figure 10, the simulation results of running different algorithms with different start and goal points are shown. The specific data comparison is given in Table 2.

From the data in Table 2, it can be seen that the number of nodes searched by the improved A-star algorithm is 92, which is 60.2% less than the number of nodes searched by the traditional A-star search node 231 in the case of the same grid environment, start point and goal point. The path length planned with the improved A-star algorithm is 51.1 m, which is 3.9% less than the path length planned with the traditional A-star algorithm. The search time of the improved A-star algorithm is 3.8 s, which is 63.8% less than the traditional A-star search time of 10.5 s. Therefore, compared to the traditional A-star algorithm, the improved A-star algorithm shows further improvement in terms of the number of search nodes, path length and search time. Meanwhile, compared to the bidirectional A-star algorithm, there is a significant improvement in the number of search nodes and the search time. However, the number of improved A-star inflection points increases significantly. Therefore, the path must be smoothed.

**Figure 11:**
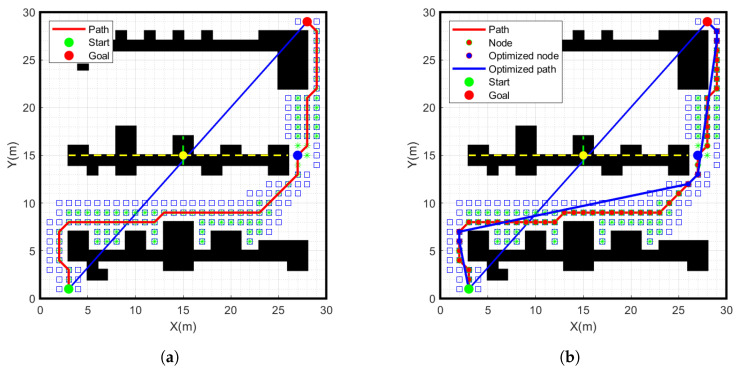
Simulation experiment results of improved A-star algorithm. (**a**) Improved A-star algorithm, (**b**) Remove redundant nodes.

As shown in Figure 11, we performed redundant node removal on the path generated by the improved A-star algorithm and obtained the blue path. This path avoids unnecessary turns, reduces path length and memory occupation effectively, and provides a more efficient path planning scheme for practical applications.

### 4.2. Simulation Comparison of Different Grid Maps

**Figure 12:**
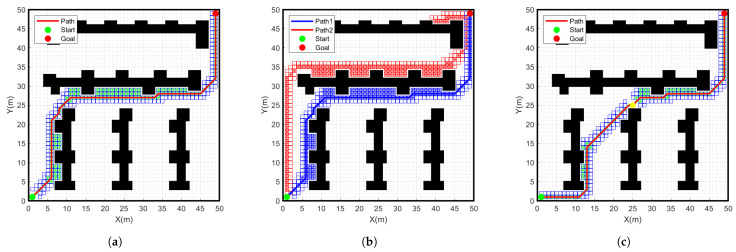
Simulation results of different algorithms for start point (1, 1) goal point (49, 49) on a 50 × 50 grid map. (**a**) Traditional A-star algorithm, (**b**) Bidirectional A-star algorithm, (**c**) Improved A-star algorithm.

**Figure 13:**
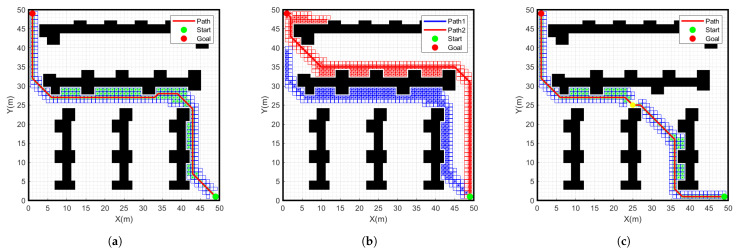
Simulation results of different algorithms for start point (49, 1) goal point (1, 49) on a 50 × 50 grid map. (**a**) Traditional A-star algorithm, (**b**) Bidirectional A-star algorithm, (**c**) Improved A-star algorithm.

To verify the effectiveness of the improved A* algorithm, we will conduct simulation experiments on different algorithms in a 50 × 50 environment map. As shown in Figure 12 and Figure 13, the simulation results of the traditional A-star algorithm, the bidirectional search A-star algorithm and the improved A-star algorithm are shown in a 50 × 50 environment map. The specific data comparison is given in Table 3.

From the data in Table 3, it can be seen that the number of nodes searched by the improved A-star algorithm is 98, which is 29.5% less than the number of nodes searched by the traditional A-star 139 in the 50 × 50 grid map. The path length planned with the improved A-star algorithm is 84.2 m, which is 3.4% less than the path length planned with the traditional A-star algorithm. The search time of the improved A-star algorithm is 4.8 s, which is 14.3% shorter than the traditional A-star search time of 5.6 s. Therefore, compared with the traditional A-star algorithm, the improved A-star algorithm has further improvement in terms of the number of search nodes, path length and search time. Meanwhile, compared to the bidirectional A-star algorithm, there is a more significant improvement in the number of search nodes and the search time. This result further verifies the feasibility of the program.

### 4.3. Simulation Experiment of Smooth Path

**Figure 14:**
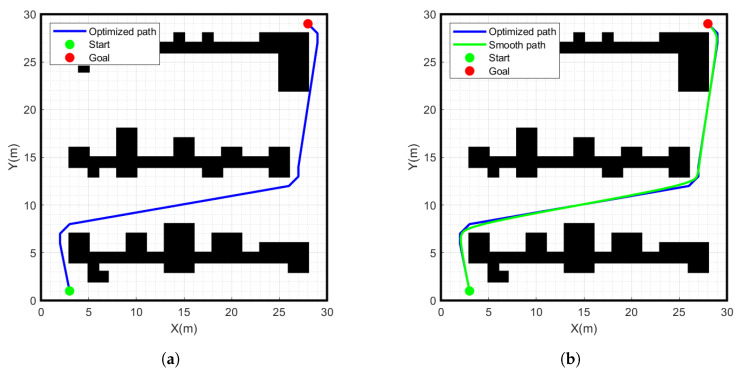
Simulation experiment results of smooth path. (**a**) Path to improve the A-star algorithm, (**b**) Cubic B-spline smooth path.

When the mobile robot makes a turn, the angle change should be as smooth as possible. Otherwise, the data are not processed in a timely manner, which can easily lead to rollover and collision of the robot. The path is processed using the cubic uniform B-spline curve, the simulation results are shown in Figure 14, where the green path shows the results of the cubic uniform B-spline curve smoothed processing. This method ensures the stability and safety of the mobile robot when turning.

## 5. Conclusions

This paper proposes an improved A-star algorithm that aims to improve the efficiency and quality of path planning. First, the evaluation function is improved by introducing the angle between the nodes and the lines connecting the start and goal points, and a bidirectional search strategy is used to start the search from the midpoint to the start and goal points simultaneously. Secondly, the generated paths are optimised by removing redundant nodes. Finally, the cubic uniform B-spline curve is introduced to smooth the planning path to eliminate sharp turns in the path. The experiment results show that the algorithm in this paper can reduce the number of search nodes, shorten the path length, shorten the search time, effectively minimize unnecessary inflection points, and guarantee the smooth operation of the robot.

Although this paper proposes an improved method, there are still some shortcomings:

1. The algorithm proposed in this paper is primarily designed for static environments and does not consider the influence of dynamic obstacles such as people in real medical testing laboratories. This limitation may cause the mobile robot to be unable to sense changes in the environment in time during the execution of the path; thus, the actual execution of the path is affected by dynamic obstacles such as people, which in turn affects the feasibility and effectiveness of navigation.
2. The algorithm proposed in this paper has only been validated through simulation experiments and theoretical analysis, without fully considering the complexities of a real medical laboratory environment. As medical testing laboratories require high stability of mobile robots, it is necessary to deploy the improved algorithm to mobile robots in real medical testing laboratories to further verify its reliability and stability.

## Figures and Tables

**Table 1:** Comparison of simulation experiment results for different heuristic functions.

Distance Formula	Number of Search Nodes	Number of Turns	Path Length/m	Time/s
Manhattan distance	67	5	50.7	2.6
Euler distance	435	9	50.7	25.8
Chebyshev distance	447	11	50.7	27.4

**Table 2:** Comparison of simulation results of different algorithms in 30 × 30 grid map.

Coordinates of Start and Goal Points	Method	Number of Search Nodes	Improving Ratio	Number of Turns	Improving Ratio	Path Length/m	Improving Ratio	Time/s	Improving Ratio
(3, 1) (28, 29)	Traditional A-star algorithm	231		5		53.2		10.5	
	Bidirectional A-star algorithm	161	30.3%	9	−80%	54.1	−1.7%	6.7	36.2%
	Improved A-star algorithm	92	60.2%	13	−160%	51.1	3.9%	3.8	63.8%
(28, 2) (3, 28)	Traditional A-star algorithm	153		8		49.1		6.0	
	Bidirectional A-star algorithm	129	15.7%	12	−50%	47.4	3.5%	5.1	15%
	Improved A-star algorithm	86	43.8%	10	−25%	47.4	3.5%	3.9	35%

**Table 3:** Comparison of simulation results of different algorithms in 50 × 50 grid map.

Coordinates of Start and Goal Points	Method	Number of Search Nodes	Improving Ratio	Number of Turns	Improving Ratio	Path Length/m	Improving Ratio	Time/s	Improving Ratio
(1, 1) (49, 49)	Traditional A-star algorithm	139		9		87.2		5.6	
	Bidirectional A-star algorithm	275	−97.8%	9		87.2		14.5	−158.9%
	Improved A-star algorithm	98	29.5%	10	−11.1%	84.2	3.4%	4.8	14.3%
(49, 1) (1, 49)	Traditional A-star algorithm	153		7		88.4		6.0	
	Bidirectional A-star algorithm	129	15.7%	12	−71.4%	93.4	−5.7%	5.1	15%
	Improved A-star algorithm	86	43.8%	10	−42.9%	85.6	3.2%	3.9	35%

## Data Availability

No new data were created or analyzed in this study. Data sharing is not applicable to this article.
